# How effective is peer-to-peer support in cancer patients and survivors? A systematic review

**DOI:** 10.1007/s00432-023-04753-8

**Published:** 2023-04-30

**Authors:** A. Kiemen, M. Czornik, J. Weis

**Affiliations:** Comprehensive-Cancer Centre Freiburg (CCCF), Medical Faculty of the Albert-Ludwigs University, Endowed Professorship for Self-Help Research University Clinic, Hugstetterstr. 49, 79106 Freiburg, Germany

**Keywords:** Peer-to-peer support, Cancer, Quality of life, Survivorship care, RCT, Psychosocial outcomes

## Abstract

**Purpose:**

Core components of peer-to-peer (PTP) support for cancer survivors include informational, emotional, and psychosocial aspects. Previous literature on peer support in cancer includes both professionally and peer-led support. Our objective was to summarize studies on the effects of non-professionally led PTP support in cancer.

**Methods:**

We performed a systematic research on studies in PTP support of adult cancer survivors with an interventional design, comparing outcomes of PTP support against any control. We included all studies with a precise definition of a PTP support, published from January 2000 up to March 2023 in peer-reviewed journals in English or German.

**Results:**

Out of *N* = 609 identified publications, we were are able to include *n* = 18 randomized-controlled trials (RCTs) fulfilling our inclusion criteria. Main settings were dyadic support via telephone, face-to-face (FTF), and web-based online support. Most common outcomes were distress, depressive symptoms, anxiety, and quality of life (QoL). Overall, we found only small effects of PTP support on depression/anxiety, coping, or sexual functioning. Beneficial effects associated with the PTP intervention were apparent in particular in BRCA, in FTF settings, and in assessments of cancer-specific QoL outcomes.

**Conclusion:**

This review shows that there are a few RCT investigating the effect of PTP support with short-term effects. Overall, there is a need for more RCTs with high methodological standards to evaluate the effectiveness of PTP support.

## Introduction

Cancer survivors who want actively contribute to their physical and psychological health often search for professional psychosocial care (Morris et al. 2014; White et al. 2020) or for peer support from others sharing their experience with cancer. Most common needs are to receive informational, emotional, and practical support to cope with the sequelae of cancer treatment (Park et al. 2019). Information and support provided by peers (i.e., cancer survivors who have recovered from cancer) based on their own experience could be helpful for patients who are at an earlier stage of treatment or recovery. In the first year after diagnosis and later, two-thirds of the patients who reported unmet needs in comprehensive cancer care wanted peer support in information-related needs, psychological care, and physical symptoms (Park et al. 2019). Peer support may support active coping behaviour, reduce anxiety, cultivate hope, and help to cope with the fear of cancer recurrence or progressions (Rini et al. 2007; Skirbekk et al. 2018). Some studies show that peer support is useful for improving quality of life (Hu et al. 2019; Walshe et al. 2020). The various formats of peer-to-peer (PTP) support facilitate group or individual interaction between the affected persons (Borgetto and Knesebeck 2009), emotional relief, and encouragement of strength and confidence, both in cancer patients and survivors (Meyer et al. 2015; Williams‐Brown et al. 2019). PTP groups are not led by professionals (e.g., doctors and therapists), but they can consult experts on specific issues if necessary. PTP groups are voluntary associations of individuals with the same disease or condition arranged at various local or regional levels of organization. These groups meet to share health-related issues and to support each other in coping with illness and illness-related distress affecting themselves or their families. It has been shown that effective social support, especially through peer support groups, is one of the influencing factors for cancer outcome in general (Kroenke 2018). Specifically, associations between low levels of social support and breast cancer mortality have been demonstrated. Frequent social contacts may help to mitigate cancer and treatment-related decrease of physical functioning. Social contacts help to normalize daily living with cancer (Guida et al. 2020). Participation in peer support groups has a preventive impact both on secondary (disease recurrence) and tertiary levels (disease impairment) (Straka 2006). Patients are equally willing to use the Internet for health information, support, and to address unmet psychosocial needs, so PTP support includes online peer support options.

Nowadays, different forms of peer support for patients at various stages of their disease or treatment have emerged. Common to all PTP interactions is the mutual support from patient to patient through sharing disease experiences, and addressing unmet psychosocial needs. However, outcomes of peer support are difficult to determine (Haines et al. 2018), and voluntary PTP support programs have received little scientific research compared to professionally led programs (Meyer et al. 2015). A major difficulty of psychosocial research in PTP support activities is the designing of appropriate randomized-controlled trials (RCTs) with standardized procedures. Blinding is not feasible and the allocation to the intervention or control group may produce bias. Patients may decline to participate if they do not receive the intervention group (Edgar et al. 2003). First studies in PTP support research have detected that PTP, mainly face-to-face and group Internet peer support programs, may promote an effective strategy for disease management support (Hoey et al. 2008). Recent research suggests that it might be the unique position of peer supporters with the non-hierarchical, reciprocal relationship between patients and peers that complement formal psychosocial support (Kowitt et al. 2019). Nevertheless, only a few studies are addressing the effectiveness of patients’ peer support. In research of various programs of peer support, quality of life (QoL), psychosocial well-being, health literature, and informational level are the most common outcomes (Haack et al. 2018; Haines et al. 2018; Toija et al. 2019). Studies on PTP interactions in patients with breast cancer (BC) presume that encountering similarly affected patients creates a safe social network that is critically important for the provision of informational and emotional support to cancer patients and their families (Meyer et al. 2015). Further, PTP interactions may have a positive short-term effect on well-being, mediated by changes in life purpose, and impact on long-term effects, including disease progression (Mens et al. 2016; Skirbekk et al. 2018; Kowitt et al. 2019).

Considering the above given definitions, PTP support settings include one-to-one (1t1) or group interactions, meetings face-to-face (FTF), via telephone or via email, online groups or chatrooms. The settings range from groups for patients with a specific cancer diagnosis or mixed cancers or together with their relatives or partners. The programs are available for patients at any stage of the disease, for newly diagnosed patients as well as for long-term survivors after cancer. The special feature of peer support is that those personally affected provide each other with psychosocial support, which is not the same as psychosocial care by professionals.

This review is aimed to analyse the evidence of non-professionally led PTP support programs for cancer survivors with respect to patient-related outcomes. The research questions of this review are ‘How effective is PTP support for cancer patients and survivors?’, and ‘Which outcomes are addressed by the studies evaluating PTP?’.

## Methods

Following the research questions, we developed a search strategy based on the PICO* criteria (Santos et al. 2007; Schardt et al. 2007) and on the key definition of PTP support. One general definition describes PTP as mutual psychosocial support by sharing experiences with others affected by the same disease without any facilitation by a health professional.

### Eligibility criteria

As inclusion criteria, references had to fulfil a precise definition of PTP support according to Johnson et al. (Johnson 2000): peer support programs bring together individuals with similar diagnoses and problems for learning from each other, sharing experience, and providing mutual support. PTP programs should be neither directed nor structured by a health professionals. This definition includes also online formats of peer support. Usually, professionals train former patients, who are typically more than 1 year post-treatment, e.g., in communication skills, or they provide up-to-date information on disease management and treatment options. In general, this trained peer support was not considered professional support. Therefore, these studies met the inclusion criteria. Further eligibility criteria were articles with a controlled interventional design, published from 01/2000 to 03/2023, in English or German language in peer-reviewed journals, with evaluation of peer support interventions for adults > 18 years with cancer of any diagnosis, any stage, any treatment, and any time since diagnosis.

Excluded were review articles, cross-sectional studies, non-interventional/observational studies, and qualitative or mix-methods studies. We further excluded studies if no definition of peer support was provided or the PTP support program was not clearly described. PTP support interventions were also excluded if health professionals were involved in initiating or leading a group.

### Search strategy and data synthesis

**Fig. 1:**
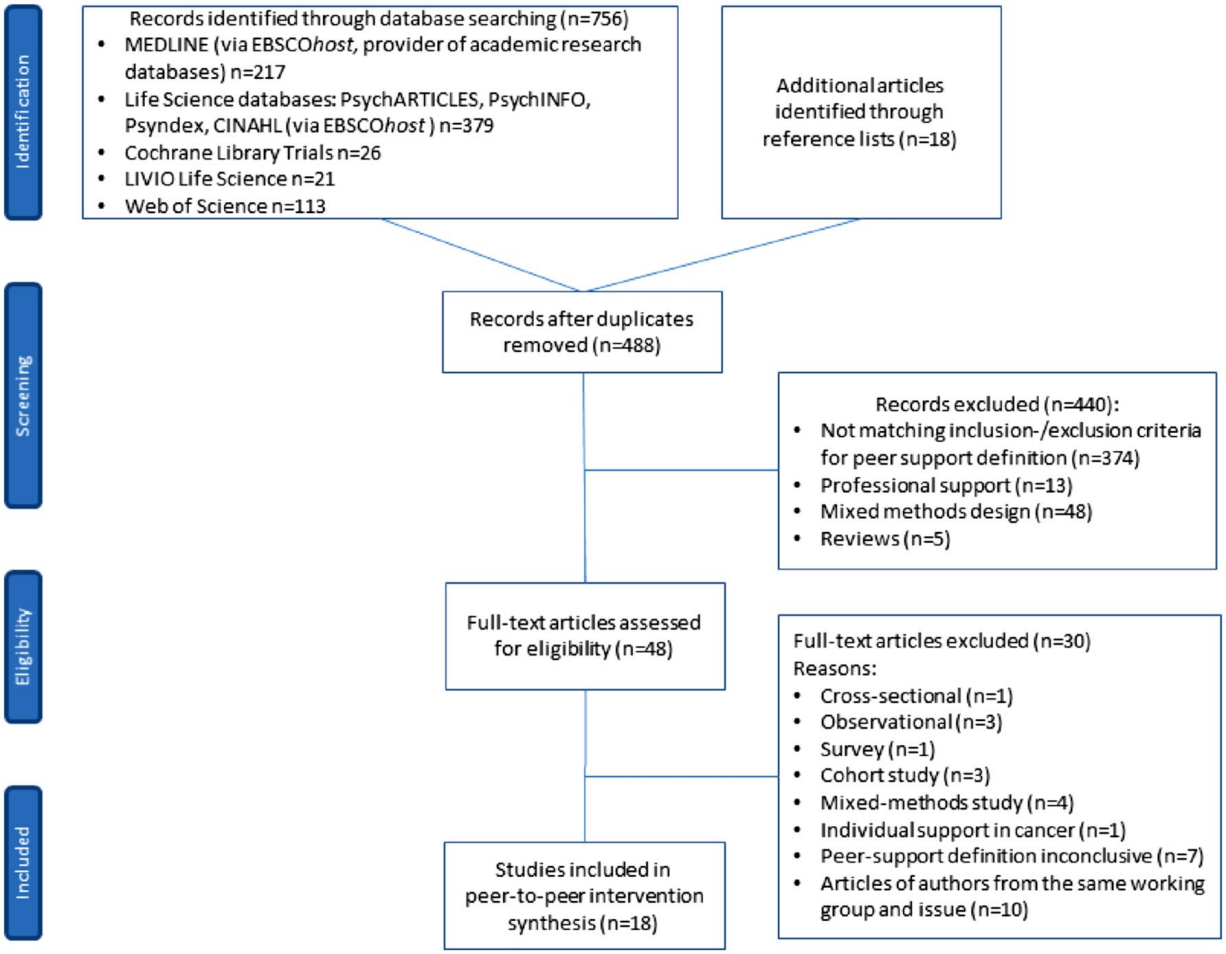
PRISMA flowchart of bibliographic literature search and study selection

Based on the specified PTP support definition and with the focus on outcomes of PTP support, this systematic literature review included an exhaustive inventory of international scientific research in the field of peer support and cancer. The critical appraisal of references from relevant reviews and reference lists of relevant studies completed the search. We used the subsequent electronic databases: Medline, PsycInfo, Psyndex, PsychArticles, CINAHL, Cochrane Library, LIVIO, and Web of Science. The search strategy combined following MeSH terms in Medline database: Word in Major Subject Heading [MJ] (cancer or neoplasms or oncology or tumour or malignancy) AND Word in Subject Heading [MW] peer group OR MW (support groups or self-help groups or peer support) AND MW quantitative* AND MW intervent* (*N* = 217 publications). We searched following key subjects in PsycInfo, PsychIndex, Psychlit, and CINAHL databases: Subject terms [SU] (cancer or neoplasms or oncology or tumour or malignancy) AND SU peer group OR SU (support groups or self-help groups or peer support) AND SU quantitative* AND SU intervent* (*N* = 379 publications). The limiters we used were academic, peer-reviewed journals; published date: 20000101–20230301; human; adults 19 + years; language: English, German. We provide this systematic literature review according to the PRISMA (Preferred Reporting Items for Systematic Reviews and Meta-Analyses, http://prisma-statement.org) guidelines for all relevant full-text articles. The disposition of references is shown in Fig. 1 [PRISMA flowchart (Moher et al. 2009)], detailing the literature search and study selection process.

Data extraction and quality assessment were conducted by two independent reviewers (AK and DB) who assessed titles and abstracts against the a priori defined study eligibility criteria: (1) focus on cancer peer support; (2) controlled interventional design; (3) published in English or German; (4) n peer-reviewed journals. We resolved disagreements through discussion with the senior author (JW) until we reached consensus. For a few references, it was difficult to clearly identify them as PTP interventions, because a definition of support was not always provided in the study method section. If provided, the definition included both PTP support without any facilitation by health care professionals and professional support, which could mean initiating a group, leading the group sessions or training peer counsellors.

There is one special feature to report about the 18 included studies: seven references have corresponding studies which are not included due to the following reasons: three references are pilot or feasibility studies of the included studies (Chambers et al. 2015; Giese-Davis et al. 2006; Weber et al. 2007a) without intervention (i.e., Chambers et al. 2013, 2013); Giese-Davis et al. 2006 (Giese-Davis et al. 2006); Weber et al. 2004 (Weber et al. 2004)). Three studies performed later subgroup analyses based on the data set of Pinto et al. 2015 (Pinto et al. 2015) (i.e., Pinto et al. 2017 evaluated effects among coaches (Pinto et al. 2017); DeMello et al. 2018 evaluated a ‘sedentary group of BC survivors compared to coaches’ (DeMello et al. 2018), and Pinto et al. 2023 examined a further second analysis of the previous data (Pinto et al. 2023). One reference used the identical data set of HØybye et al. 2010 (Høybye et al. 2010) to identify the social and psychological characteristics of Internet support-group participants versus non-participants (Hoybye et al. 2010).

### Study quality assessment

**Fig. 2:**
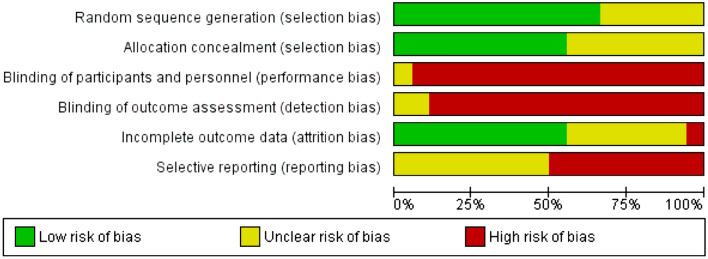
Risk-of-bias graph: review authors’ judgements about each risk-of-bias item presented as percentages across all included studies

**Fig. 3:**
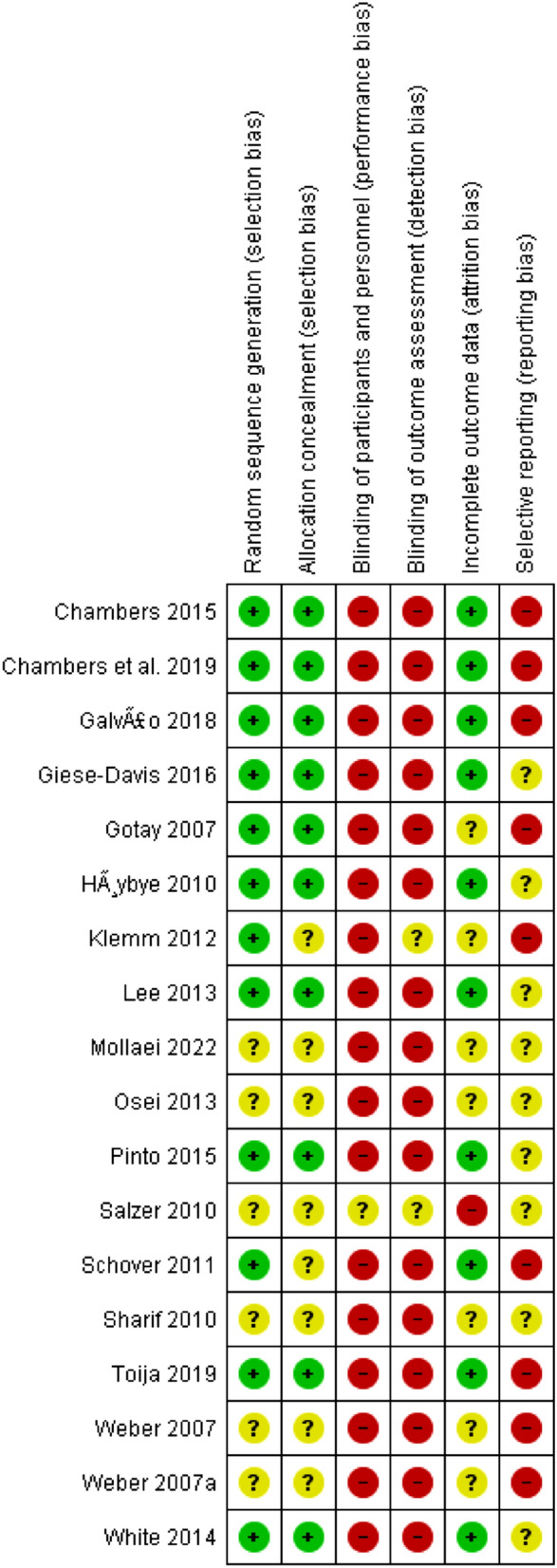
Risk-of-bias summary: review authors’ judgements about each risk-of-bias item for each included study

To perform the best evidence synthesis, we used Cochrane’s Risk of Bias Tool [RoB-2, (Sterne et al. 2019)] to rate the quality of the studies according to the suggested classification as ‘low’, ‘unclear’, or ‘high’ risk of bias. Elements evaluated were random sequence generation [selection bias], adequate allocation concealment [selection bias], blinding of participants and personnel [performance bias], blinding of outcome assessors [detection bias], incomplete outcome data [attrition bias], and selective reporting [reporting bias]. The nature of the study designs precludes blinding of participants, so this criterion can be assessed but not taken into account. However, the RoB analysis has demonstrated a between-trial heterogeneity. The RoB judgements are presented in Figs. 2 and 3 in the Appendix.

## Results

### Selection of studies

Database searches identified 756 hits. Another additional 18 publications were identified through reference lists. After removing duplicates, 488 references remained. Following the review of the abstracts and titles, 440 articles did not meet the inclusion criteria and were eliminated. The remaining 48 full-text articles were assessed for eligibility. This led to the elimination of additional 30 articles, and 18 studies remained.

### Study characteristics and study quality

All 178 studies included were RCTs. The study characteristics are presented in Table 1. The majority of patients were women with breast cancer (BC, *n* = 10 studies), followed by prostate cancer (*n* = 6 studies) and two trials considering mixed cancer diagnoses. Almost all studies included one treatment group (TG) compared with a control group (CG), which was care as usual. Only one study investigated two treatment arms (PTP support and nurse support) compared with care as usual (Chambers et al. 2015). In most of the RCTs, professionals prepared the former patients, who were typically more than 1 year post-treatment, to conduct as peers the peer-support programs, e.g., communication skills or information on disease management. This training of the peers was not considered as professional support. Although one of the interventional arms included professional support, we did not exclude this study, because the other arm assessed non-professional support. The overall RoB rating demonstrates a mixed quality of the studies included. Table 1 presents the list of the *N* = 18 included studies, and a summary for each study evaluating PTP support outcomes from January 2000 to March 2023 according to the PICO (P = participants, I = intervention, C = comparator, and O = outcome) elements. We present effect measures for each outcome (i.e., risk ratio, mean difference, and effect sizes) as reported.

**Table 1:** Summary of study characteristics and outcomes

#	Reference	Sample Size TG = treatment group CG = control group	Primary endpoint(s)	Participants/cancer	Intervention		Comparator	Outcome	
					Peer aspect	Support setting		Baseline (BL) characteristics	Results (0) = no effect (+ +) = sign effect (–) = contrary effect
1	Gotay et al. (2007)	*N* = 305 total *n* = 152 TG *n* = 153 CG	Psychosocial distress [CARES-SF]; Depression [CES-D score]	Breast cancer recurrence	Assistance cancer survivors provide to other patients; counsellors were ≥ 1 year post-recurrence were trained and monitored; had to score in a non-depressed range on the CES-D (≤ 16)	Dyadic (1t1) telephone-delivered	TG versus CG (care as usual); CG patients were mailed materials that TG patients had received previously	Generally well-balanced for prognostic factors, except: TG received more CTx^1^, CG received more HT^2^ for recurrence; in CG more patients progressed during 6 months on study	(0) TG vs. CG at 3 months: multivariate analysis; psychosocial distress (OR 0.80; 95% CI 0.41–1.54; *p* = 0.50); (0) Depression (OR 1.38; 95% CI 0.80–2.37; *p* = 0.24)
2	Chambers et al. (2015)	*N* = 189 total hetero-sexual couples; *n* = 62 nurse TG *n* = 63 peers’ TG *n* = 64 CG	Psycho-sexual morbidity: Sexual adjustment in men (International Index of Erectile Function) and women (Female Sexual Function Index); unmet sexual supportive care needs; masculine self-esteem; marital satisfaction; utilisation of erectile aids	Prostate cancer	Peer support volunteers (counsellors, *n* = 15) were prostate cancer survivors ≥ 12 months post-prostatectomy, received 12 h of training	Dyadic (1t2) telephone-delivered	TG1: peer-delivered TG2: nurse-delivered couples-based telephone support; CG: standard medical care + set of patient education materials	No significant differences between the study groups at BL on outcomes or socio-demographics. TG2 had significantly longer sessions compared with the TG1, *t*(806) = 6.79, *p* < 0.001	(0) No significant effects on psycho-sexual morbidity for men or women TG1 and TG2 were more likely to use medical treatment for erectile dysfunction than CG (z = 2.41; *p* = 0.016; *z* = 2.64; *p* = 0.008)
3	Chambers et al. (2019)	*N* = 107 male patients *N* = 91 female partners completed 5-year assessment	Details in (Chambers et al. 2015); assessed at 2-, 3-, 4-, 5-year follow-up	Prostate cancer	Details in (Chambers et al. 2015)	Details in (Chambers et al. 2015)	Details in (Chambers et al. 2015)	No significant differences in age, education level, income level, length of time married, and marital satisfaction at BL between participants who were retained in the study at 5 years and those who had withdrawn	Sexual adjustment: (0) No significant group difference for men at any time; (+ +) Higher in women of TG1 at 2 and 3 years (*z* = 3.17, *p* = 0.002, *z* = 2.94, *p* = 0.035) Sexual supportive care needs: (–) Higher in men of TG1 at 3 years (*z* = 3.34, *p* = 0.001); (–) Higher in women of TG1 at 2 and 3 years (*z* = 2.07, *p* = 0.038; *z* = 3.46, *p* = 0.001); Masculine self-esteem: (–) Less in TG1 than TG2 at 2 and 5 years (*z* = 1.94, *p* = 0.052; *z* = 2.01, *p* = 0.045); Martial satisfaction: (–) Less in women of TG1 than CG (*z* = 2.80; *p* = 0.005) and TG2 (*z* = 2.74; *p* = 0.006) at 4 years: Treatment for erectile dysfunction: (–) TG1 and TG2 were more likely to use medical treatment for erectile dysfunction at 2, 3, 4, and 5 years (*z* = − 2.88; *p* = 0.060; *z* = − 2.05; *p* = 0.040, *z* = − 3.13, *p* = 0.002; *z* = − 2.84, *p* = 0.005)
4	Galvão et al. (2018)	*N* = 463 total *n* = 232 TG *n* = 231 CG	Compliance with exercise guidelines in a multimodal peer-led intervention to increase exercise participation	Prostate cancer	Peers were experienced in community support	Group peer support, telephone-delivered (via teleconference)	TG versus CG; TG: self-management materials and monthly telephone-group peer support led by 2 peer support volunteers for 6 months CG: standard medical care + a set of education material	Patients on average 8 months post‐diagnosis (*SD* = 3.0, range = 0.6–21.6 months). No sig diff on any demographic or psychosocial variable, except for age: men in TG were significantly younger (*p* = 0.05) (*M* = 63.7 years, *SD* = 7.6) than men in CG (*M* = 65.1 years, *SD* = 7.8)	(+ +) Significant interaction between time × study group for resistance exercise duration (*p* = 0.048 [95% CI 0.15–31.07]) at 3 months; Post-hoc differences: TG greater resistance at 3 months (19.4 [95% CI 6.52–32.28] min/week, *p* = 0.003) and 6 months (14.6 [95% CI 1.69–27.58] min/week, *p* = 0.027)
5	Giese-Davis et al. (2016)	*N* = 104 total *n* = 52 TG *n* = 52 CG	Cancer-specific QoL [FACT-B: BSW] and trauma symptoms [PCL-C]; to test the hypothesis, that patients experience of their cancer as traumatic stressor moderates results	Breast cancer; newly diagnosed (≤ 3 months) at all stages of disease	30 peer counsellors, post-treatment, at least 1-year post-diagnosis with BC^3^, trained and supervised, who represented a spectrum of socioeconomic status, ethnicity, diagnosis, and treatment	Dyadic (1t1) telephone-delivered or via, email	TG versus CG TG: match with a navigator with weekly contacts up to 6 months versus CG: no further intervention (no further description of CG)	BL balanced except for age: women in TG were significantly older (*p* = 0.06, Median = 55.3, range = 33.9–80.0) vs CG (Median 51.7, range 36.5–78.8)	(+ +) QoL [FACT-BSW total score] group × time *p* = 0.02 [95% CI 0.46–1.24] improved significantly more for patients in TG vs. CG at 12 months
						or FTF for up to 6 months			
6	Høybye et al. (2010)	*N* = 794 total *n* = 361 TG *n* = 433 CG	Psychological distress [POMS-SF] and Adjustment to cancer [Mini-MAC]: To assess the incremental effect of participation in an ISG^4^ following a week-long rehabilitation program: differences in changes	All cancers (different stages and treatments)	No therapeutic content or information services were offered within the self-guided space for communication	Web-based/online support	TG versus CG TG: Internet peer support group and 2 h introductory lecture for internet use versus CG: No treatment beyond ordinary rehabilitation programme and 2 h recreational time with no specific programme	Demographic characteristics in the two conditions at BL were significantly different, and the groups assigned to intervention were containing more men, younger persons, and more cohabiting persons	(0) No effect of the intervention on total mood disturbance or on any of the POMS subscales at any time, except for a transient difference on the subscales confusion/bewilderment (*p* = 0.001) and depression/dejection (*p* = 0.04) at the 6-month follow-up (0) No significant differences on mental adjustment to cancer, and self-rated health at 12 months post-intervention
7	Klemm (2012)	*N* = 50 total *n* = 24 TG *n* = 26 CG	Depressive symptoms (CES-D)	Breast cancer	Peer-led online support run by the subjects themselves without use of preselected topics or input from a moderator	Web-based/online support	TG versus CG peer-led online support versus moderated online support	BL difference between arms in depressive symptoms (CES-D scores) with the peer-led group having significantly higher scores (16.01 vs. 8.96; *t*[128.42] = 1.99, *p* = 0.049) than the moderated group	(0) No significant effect for group (moderated vs peer-led), time, or time × group at 12 weeks: CES-D 9.88 vs 10.19 for peer-led group vs. moderated group (*p* < 0.05)
8	Lee et al. (2013)	*N* = 129 total *n* = 64 TG *n* = 65 CG	Self-efficacy [self-efficacy scale], Anxiety and depression [HADS], and Mental adjustment [Mini-MAC]	Breast cancer; newly diagnosed; within 1 month of diagnosis	Disease-free survivors of BC at least 1 year after primary cancer therapy and trained to act as dyadic support partners	Dyadic (1t1) telephone-delivered or dyadic FTF in person	TG versus CG TG: At least four meetings once a week during the 6-week period after surgery versus CG = Standard care	Sociodemographic and clinical characteristics did not differ significantly between the two groups	(+ +) Greater increase in self-efficacy for self-management (*p* = 0.043) in TG; (0) No significant differences in other variables
9	Mollaei et al. (2022)	*N* = 80 total *n* = 40 TG *n* = 40 CG	Resilience [CD-RISC, Connor-Davidson Resilience Scale], at baseline, one week, and three months after intervention	Patients with cancer	Cancer survivors; Peer group comprised a woman with breast cancer, a woman with uterine cancer, a man with Hodgkin’s cancer, a man with colorectal cancer; they all underwent self-care training and were assigned to educational topics	FTF 1 to group of ten	TG versus CG TG: peer group members trained the intervention group (self-care education) in four sessions (1x/week) over 6 weeks CG: routine care	No significant difference between groups in terms of demographic and clinical information	(+ +) Significant difference one week after intervention (*p* = 0.01); group × time interaction (*F* = 6.47, *p* = 0.004) indicating higher increase in resilience score over time in TG (–) No significant difference at three months (*p* = 0.11)
10	Osei et al. (2013)	*N* = 40 total *n* = 20 TG *n* = 20 CG	Quality of life [SF-12; EPIC-26 (Extended Prostate Cancer Index); Satisfaction with life scale; Relationship satisfaction scale; Program satisfaction]	Prostate cancer within the previous 5 years	Website offers information, to help make informed decisions on prostate cancer testing, active surveillance, treatment options, and management of side-effects “Gain direct access to an online information exchange with others who share an interest in specific topics within the prostate cancer online community.”	Web-based/online support	TG versus CG TG: participated at least 3x/week during 6 weeks in the network program; CG = were provided with resource kits (pamphlets, including future treatment options, potential side-effects, approaches to deal with side-effects, other information	No significant difference in age between groups	(+ +) Significant interaction time × group (*F* = 2.37, *df* = 20, *p* = 0.036); improvement in 3 of 6 statistically sig areas: urinary irritation and obstruction health, sexual health, and hormonal health, with these scores returning to baseline level at 8 weeks; CG had dropped on 6 QoL measures, but also returned to baseline by 8 weeks
11	Pinto et al. (2015)	*N* = 76 total *n* = 39 TG *n* = 37 CG	Self-reported activity of MVPA^5^ (moderate-to-vigorous intensity PA)	Breast cancer	Peer support volunteers (*n* = 18) were BC survivors; were trained and supervised	Dyadic (1t1) telephone-delivered: [weekly calls up to 12 weeks in TG and CG]	TG versus CG TG = contact control + PA^6^; CG = Contact control Contact control group was asked not to change current level of activity during next 12 weeks	No between-group differences in BL characteristics (*p* = 0.05) or in BL psychosocial constructs	(+ +) Significant between-group differences at both 12 and 24 weeks: Self-reported MVPA 103.0 min/week (*SD* = 15.4, *p* < 0.001) and 34.7 min/week (*SD* = 15.5, *p* = 0.03). Mean difference accelerometer MVPA data 48.5 min/week at 12 weeks (*SE* = 11.9) and 38.7 min/week (*SE* = 12.0) at 24 weeks
12	Salzer et al. (2010)	*N* = 78 total *n* = 51 TG *n* = 27 CG	Anxiety/Depression [Hopkins Symptoms Checklist (HSCL-25)] QoL [FACT-B]	Breast cancer, newly diagnosed with Stage I or II (≤ 12 months)	Unmoderated internet peer support group (i.e., no professional facilitator)	Web-based/online support	TG versus CG Internet peer support (TG) vs Internet educational control condition	Women in the control condition had marginally worse scores on the IES at BL (*p* = 0.07; *F* = 3.48); no differences on the other dependent variables	(–) CG performed better: HSCL-25: Time × condition (*p* = 0.005; *F* = 3; *df* = 76): CG did marginally better at 4 months (score diff *d* = − 0.20; *p* = 0.10; *t* = − 1.66; df = 76; *es* = 0.40) and 12 months (*d* = − 0.19; *p* = 0.11; *t* = − 1.62; *df* = 76; *es* = 0.40) (–) Contrary to hypothesis: FACT-B Time × condition (*p* = 0.004; *F* = 6.09; *df* = 76) CG had better scores at 4 months (*d* = 9.17; *p* = 0.05; *t* = 1.98; *df* = 76; *es* = 0.48) and 12 months (*d* = 10.89; *p* = 0.03; *t* = 2.21; *df* = 76; es = 0.55)
13	Schover et al. (2011)	*N* = 300 total *n* = 152 TG *n* = 148 CG	Spiritual Well Being Functional Assessment of Cancer [FACIT-Sp]; Emotional Distress [Brief Symptom Inventory-18, BSI-18, including subscales for depression/anxiety], Female sexual function Index [FSFI]; Dyadic Adjustment Scale [A-DAS] for married women; Dating Subscale of Cancer Reha Evaluation System [CARES] for not-married women; menopausal symptoms	Breast cancer	African BC survivors, trained; *N* = 27	Dyadic (FTF) in-person versus telephone-delivered	TG versus CG TG: Full PTP (60–90 min.) incl. SPIRIT workbook versus CG: Brief telephone counselling (≤ 30 min.) including SPIRIT workbook initiated by participant	The two groups did not differ significantly in demographic and medical factors	(+ +) Greater decrease in depression scores in TG (time × treatment; *p* = 0.0362) at 6 weeks and 6 months, but no difference at 12 months (0) No sig change (time × treatment) in FSFI total score or subscale scores, A-DAS, or CARES, distress about childbearing issues
14	Sharif et al. (2010)	*N* = 99 total *n* = 49 TG *n* = 50 CG	QoL [EORTC-QLQ-C20], [EORTC-QLQ-BR23]	Breast cancer post-mastectomy (≥ 1 year after their mastectomy, completed chemo- and radiotherapy, and currently under hormone therapy)	Patients who were in stages I and II with at least 5 year remission post-mastectomy, trained	Dyadic (FTF); one peer-counsellor in front of a group of nine patients	TG versus CG TG: Peer educator conducted four 1 h sessions on a weekly basis for one month versus CG: No intervention, educational pamphlet after the last stage of data collection	TG and CG were similar regarding age, marital status, educational level, and in QoL^7^ scores at BL	(+ +) Significant improvement in TG and reduction in CG: QoL (time × group; *p* = 0.001) including global health (TG 80.0 ± 17.9 vs. CG 61.66 ± 21.8 at 2 months), role-, cognitive-, emotional-, and social-functioning; physical functioning *p* = 0.041; (+ +) Significant decline in four symptomatic aspects: fatigue, pain, insomnia, and loss of appetite in TG and increase in CG (+ +) Increase of all QlQ-BR23 aspects in TG: body image, sexual function, sexual satisfaction, and future perspectives (*p* < 0.001); decrease in CG
15	Toija et al. (2019)	*N* = 260 total *n* = 130 TG *n* = 130 CG	HRQoL [15D, and EORTC QLQ-C30; EORTC QLQ-BR23]	Breast cancer, newly diagnosed (between diagnosis and beginning of treatment)	Survivors (< 2 years), trained, volunteer; *N* = 15	Dyadic (1t1) telephone-delivered	TG versus CG TG: peer support via telephone one-to-five times during the first weeks after diagnosis versus CG: Usual care	TG and CG were similar regarding age, marital status, number of children, educational level, employment status, professional status BL QLQ-C30 and QlQ-BR23 were similar in TG and CG, but for the QLQ-BR23, TG had slightly but sig. (*p* < 0.05) more breast and arm symptoms	(0) 15D BL to 3 months: Significant deterioration in the whole sample (0.92, *SD* = 0.07 to 0.89, *SD* = 0.09; *p* < 0.001): TG, 0.029 vs. CG 0.044, difference not significantly. During follow-up, small differences in some dimensions between groups disappeared EORTC-QLQ-C30, EORTC-QLQ-BR23: no consistent meaningful differences
16	Weber et al. (2007b)	*N* = 72 total *n* = 37 TG *n* = 35 CG	QoL: HRQoL [MOS SF-36]; Prostate-spec QoL [UCLA]	Prostate cancer, 6 weeks after surgery	Former patients who had had radical prostatectomy at least 3 years prior to the study	Dyadic (FTF) in person	TG versus CG TG: dyadic peer support + usual health care CG: Usual health care	No significant differences on physical or emotional general QoL between groups at baseline; for prostate cancer-specific QoL, the TG had significantly better BL urinary and bowel function than CG	(0) QoL measures with SF-36 were significantly skewed and could not be evaluated as expected (+ +) UCLA: physical role functioning (*p* = 0.01), bowel function (*p* = 0.03), mental health (*p* = 0.01), social function (*p* = 0.04)
17	Weber et al. (2007a)	*N* = 72 total *n* = 37 TG *n* = 35 CG	Depressive Symptoms [Geriatric Depression Scale, GDS]; Self-Efficacy [Stanford Inventory of Cancer Patient Adjustment, SICPA]	Prostate cancer, 6 weeks after surgery	Former patients who had had radical prostatectomy at least 3 years prior to the study	Dyadic (FTF) in person	TG versus CG TG: dyadic peer support + usual health care CG: Usual health care provided by the urologist	No significant differences between groups on demographic characteristics or study outcome variables	(+ +) TG: significantly lower depression (*M* = 2.53, *SD* = 3.662, and *M* = 0.92, SD = 1.320; *t* = 2.424, *p* = 0.020) (+ +) TG: significantly higher self-efficacy (*M* = 300, *SD* = 43.763, and *M* = 328.89, *SD* = 40.630; *t* = − 2.905, *p* = 0.005) at 8 weeks
18	White et al. (2014)	*N* = 207 total *n* = 105 TG *n* = 102 CG	Distress [Impact of Event Scale, IES]	Breast cancer with BRCA1 or BRCA2 mutation	Trained peer volunteers, also BRCA1/2 mutation carriers who were within 5 years of receiving mutation status notification;	Dyadic (1t1) telephone-delivered	TG versus CG TG: Peer matching based on cancer and surgery history, age, marital status, and children; telephone contacts 6 × over 4 months versus CG: Usual care	BL demographic and clinical characteristics of participants in the TG and CG were similar	(+ +) Greater decrease in distress scores in the TG than CG: at time 2 (mean difference − 5.96; 95% CI − 9.80 to − 2.12; *p* = 0.002) and at time 3 (mean difference − 3.94; 95% CI, − 7.70 to − 0.17; *p* = 0.04)

^1^CTx = Chemotherapy

^2^HT = Hormone therapy

^3^BC = Breast cancer

^4^ISG = Internet-based support group

^5^MVPA = Moderate-to-vigorous physical activity

^6^PA = Physical activity

^7^QoL = Quality of Life

### Tables of results

Overall, the 18 studies investigated as primary outcomes distress (*n* = 4), depressive symptoms and anxiety (*n* = 6), health-related QoL (*n* = 7), and resilience (*n* = 1). Further, mainly secondary psychosocial outcomes include resilience scores, coping strategies (i.e., mental adjustment to cancer) and self-efficacy (SE, *n* = 4), physical activity (PA, *n* = 2), trauma symptoms (*n* = 1), and sexual functioning (*n* = 3). We found three different forms of PTP support settings in the included trials: (1) dyadic face-to-face (FTF) counselling (*n* = 6, including two studies combining FTF with telephone-delivered support or support via email); (2) telephone-based counselling in various settings [dyadic one-to-one (1t1) (*n* = 8), or one-to-two couples-based (*n* = 1), or two-to-group setting (*n* = 1]); and (3) web-based interventions [online support groups (OSG) *n* = 4]. Intervention studies investigated the FTF setting compared to care as usual (11 of 12 trials), except for one study (Schover et al. 2011) which used a brief telephone counselling initiated by the participant as a control condition. Regarding the CG in the 4 OSG, information on cancer-related websites or written educational material were given, except for (Klemm 2012) who used a moderated OSG by social workers as CG compared to a peer-led OSG.

### Synthesis of results

In *n* = 13 out of 18 studies, the PTP intervention was significantly associated with one of the study outcomes (4 × distress, resilience and coping; 3 × depression/anxiety; 4 × QoL scores; and 2 × physical activity scores), and the effect varied in sustainability. Significant short-term effects of the PTP intervention were identified on distress (White et al. 2014), resilience (Mollaei et al. 2022) and self-efficacy (Weber et al. 2007a; b; Lee et al. 2013), short-and long-term effects on depressive symptoms and anxiety (Weber et al. 2007a; Lee et al. 2013), on QoL scores (Weber et al. 2007b; Sharif et al. 2010; Osei et al. 2013; Giese-Davis et al. 2016), and on physical activity (Pinto et al. 2015; Galvão et al. 2018).

Seven studies assessed distress and coping strategies (Gotay et al. 2007; Høybye et al. 2010; Schover et al. 2011; White et al. 2014) and (Weber et al. 2007a; Høybye et al. 2010; Lee et al. 2013) using seven different outcome instruments (Care-SF, IES, BSI-18, POMS-SF, Mini-MAC, and 2 × self-efficacy [SE-Scale, and SICPA]) (Table 2a). One of these reported a significant decrease in distress scores in TG compared to CG. The IES tool was used revealing a mean score difference of − 5.96 ([95% CI − 9.80 to − 2.12], *p* = 0.002) at 4 months immediately after the end of a dyadic telephone-delivered intervention and − 3.94 ([95% CI − 7.70 to − 0.17], *p* = 0.04) at 6 months (i.e., 2 months after intervention) in female *BRCA1/2* mutation carriers provided by peer-*BRCA 1/2* mutation carriers (White et al. 2014). In two studies, there was a significant change in SE for the TG: in one study, SE increased at 6 weeks after the 1t1 telephone-delivered support in patients with BC (*p* = 0.043) (Lee et al. 2013). In another study, SE increased at 8 weeks after an FTF support in patients with prostate cancer (M(± SD) = 300(± 43.76), and 328.9(± 40.63); *t* = − 2.905, *p* = 0.01) (Weber et al. 2007a). Cancer patients’ resilience improved significantly with peer-counselling in a group of patients 1 week after the intervention compared to the control group (*F* = 15.58, *p* < 0.001) (Mollaei et al. 2022).

**Table 2:** Outcomes by PTP support setting

a) Distress, resilience, and coping by PTP support setting													
Outcome	Study characteristics	1t1					FTF				OSG		
		(−)	(0)	(0)	**(+ +)**	**(+ +)**	(−)	(0)	**(+ +)**	**(+ +)**	(−)	(0)	(+ +)
Distress/coping	Author	⇓Reading direction	Gotay et al. (2007)	Lee et al. (2013)	White et al. (2014)	Lee et al. (2013)	⇓Reading direction	Schover et al. (2011)	Mollaei et al. (2022)	Weber et al. (2007b)	⇓Reading direction	Høybye et al. (2010)	
	Endpoint and assessment tool		Psychosocial distress [CARES-SF]	Adjustment to Cancer [Mini-MAC]	Distress [Impact of Event Scale, IES]	Self-Efficacy for self-management [SE-Scale]]		Emotional distress/Brief Symptom Inventory-18, [BSI-18]	Resilience Score [CD-RISC]	Self-Efficacy [SICPA]		Psychological distress [POMS-SF] and [Mini-MAC]	
	Time of assessment		Short term (primary, at 3 months)	Short-term (6 weeks)	Short term (4 months [t2, end of intervention), 6 months [t3, at 2 months])	Short-term (6 weeks)		Short-term/long-term (6 weeks/6, and 12 months)	Short-term (1 week)	Short-term (8 weeks)		Short-term/long-term (1/6, and 12 months)	
	Duration of intervention		4–8 telephone calls over 1 months	1 meeting/week for 6 weeks	4 months	1 meeting/week for 6 weeks		3 × meetings throughout a 6 weeks treatment period	1/week for 4 weeks	1/week for 8 weeks		Up to 13 months	
	Effectiveness		No effect of the intervention	No effect of the intervention	Greater decrease in the TG than CG at t2 and t3	Significant difference in change: greater increase in TG after intervention		No effect (time × treatment group)	Significantly higher resilience score in TG at 1 week	Significantly higher self-efficacy in TG at 8 weeks		No effect of the intervention	
	Effect size		No data on effect sizes	No data on effect sizes	No data on effect sizes	No data on effect sizes		Cohen *d* 0.14	No data on effect sizes	No data on effect sizes		No data on effect sizes	

b) Depression/anxiety by PTP support setting												
Outcome	Study characteristics	1t1	1t1	1t1 or FTF	1t1	FTF				OSG		
		(−)	(0)	(0)	(+ +)	(−)	(0)	**(+ +)**	**(+ +)**	**(−)**	(0)	(+ +)
Depression/anxiety	Author	⇓Reading direction	Gotay et al. (2007)	Lee et al. (2013)		⇓Reading direction		Schover et al. (2011)	Weber et al. (2007a)	Salzer et al. (2010)	Klemm (2012)	
	Endpoint and assessment tool		Depression [CES-D]	Anxiety and Depression [HADS]				BSI-18—Depression subscale	Geriatric Depression Scale [GDS]	Hopkins Symptoms Checklist; Anxiety and Depression [HSCL-25]	Depression [CES-D]	
	Time of assessment		3 months (primary) and 6 months	6 weeks after intervention				Short-term/long-term (6 weeks/6 and 12 months)	Short-term (8 weeks)	Short-term/long-term (4/12 months)	6, 12, and 16 weeks (i.e., 4 weeks after end of intervention)	
	Duration of intervention		4–8 telephone calls over 1 months	1 meeting/week for 6 weeks				3 × meetings throughout a 6 weeks treatment period	1/week for 8 weeks	N/A	12 weeks	
	Effectiveness		No effect of the intervention	No effect of the intervention				Greater decrease in TG at 6 weeks and 6 months, but no difference at 12 months	Significantly lower depression in TG at 8 weeks	Contrary to hypothesis: CG performed better	No effect of time, or time × intervention	
	Effect size		No data on effect sizes	No data on effect sizes				Cohen *d* 0.14	No data on effect sizes	Effect size es = 0.40 at 4 and 12 months	No data on effect sizes	

c) (Health-related) quality of Life by PTP support setting											
Objective	Study characteristics	1t1		1t1 or FTF	FTF				OSG		
		(−)	(0)	**(+ +)**	(−)	(0)	**(+ +)**	**(+ +)**	(−)	(0)	**(+ +)**
(Health-related) QoL	Author	⇓Reading direction	Toija et al. (2019)	Giese-Davis et al. (2016)	⇓Reading direction	Schover et al. (2011)	Sharif et al. (2010)	Weber et al. (2007b)	Salzer et al. (2010)	⇓Reading direction	Osei et al. (2013)
	Endpoint and assessment tool		[15D, and EORTC QLQ-C30; EORTC QLQ-BR23]	[FACT-B; Breast Cancer spec well-being, BSW]		Spiritual well-being [FACIT-Sp]	EORTC-QLQ-C30], [EORTC-QLQ-BR23]	[MOS SF-36] and Prostate-specific QoL (UCLA)	Functional Assessment of Cancer Therapy-BC [FACT-B]		[SF-12; EPIC-26 (Extended Prostate Cancer Index)]
	Time of assessment		Short-term/long-term (3-/6-, and 12 months	Long-term (12 months)		Short-term/long-term (6 weeks/6 and 12 months	Short-term (4 weeks, 2 months)	Short-term (8 weeks)	Short-term/long-term (4/12 months)		Short-term (6, and 8 weeks)
	Duration of intervention		approximately 1 call/week one-to-five times	Up to 6 months		3 × meetings throughout 6 weeks	1 meeting/week up to 4 weeks	1/week for 8 weeks	N/A		6 weeks
	Effectiveness		No effect of the intervention	Significantly improvement in TG vs. CG		No effect of the intervention	Significant improvement in TG; reduction in CG	TG significantly accounted for physical role functioning, bowel function, mental health, and social function	Contrary to hypothesis: CG had better scores than TG		Significant interaction time × group at 6 weeks; return to BL level at 8 weeks
	Effect sizes		No data on effect sizes	Cohen’s *d* = 0*.*41		No data on effect sizes	No data on effect sizes	No data on effect sizes	*es* at 4 months 0.48 *es* at 12 months 0.55		No data on effect sizes

d) Other outcomes by PTP support setting												
Objective	Study characteristics	1t1					FTF			OSG		
		(−)	(0)	(0)	**(+ +)**	**(+ +)**	(−)	(0)	(+ +)	(−)	(0)	(+ +)
Others	Author	Chambers et al. (2019)	Chambers et al. (2015)	Giese-Davis et al. (2016)	Pinto et al. (2015)	Galvão et al. (2018)	⇓Reading direction	Schover et al. (2011)				
	Endpoint and assessment tool	Psycho-sexual morbidity	Psycho-sexual morbidity	Post-traumatic stress disorder Checklist-Civilian Version [PCL-C]	MVPA (moderate-to-vigorous physical activity)	Physical activity: Resistance exercise duration		Sexual Functioning [FSFI]				
	Time of assessment	Long-term (2, 3, 4, 5 year follow-up)	Short-term/long-term (3/6, and 12 months)	Long-term (12 months)	Short-term/long-term (3/6 months)	Short-term/long-term (3/6, and 12 months)		Short-term/long-term (6 weeks/6, and 12 months				
	Duration of intervention	6–8 telephone-delivered support up to 22 weeks post recruitment	6–8 telephone-delivered support up to 22 weeks post recruitment	1x/week up to 6 months	12 weeks	1x/months; 6 months		3 × meetings throughout a 6 weeks treatment period				
	Effectiveness	Sexual supportive care needs: Higher in men of TG1 at 3 years Masculine self-esteem: Less in TG1 than TG2 at 2 and 5 years Treatment for erectile dysfunction: TG1 and TG2 were more likely to use medical treatment for erectile dysfunction at 2, 3, 4, 5 years	No significant effects on sexual function, sexuality needs, sexual self-confidence, masculine self-esteem, marital satisfaction or intimacy either for men or women. Men in TG were more likely to use medical treatment for erectile dysfunction	No effect of the intervention	Significant between-group differences: Greater increase of MVPA in TG at 12 and 24 weeks	Significant interaction time × group: Greater resistance in TG at 3 and 6 months		No effect of the intervention				
	Effect sizes	No data on effect sizes	No data on effect sizes	No data on effect sizes	No data on effect sizes	No data on effect sizes		No data on effect sizes				

(+ +) = Statistically significant difference TG vs. CG at *p* < 0.05; (−) = Statistically significant difference CG vs. TG at *p* < 0.05; (0) = No significant difference between groups

*OSG* Online Support Group, *1t1* one-to-one, *FTF* face-to-face, *TG* Treatment Group, *CG* Control Group, *FU* follow-up after end of intervention, *short-term FU* (0 ≤ 6 months), *long-term FU* (≥ 6 months)

In six studies assessing depressive symptoms and anxiety (Gotay et al. 2007; Weber et al. 2007a; Salzer et al. 2010; Schover et al. 2011; Klemm 2012; Lee et al. 2013), five different outcome instruments (BSI-18, 2 × CES-D, GDS, HADS, and HSLC-25) were used (Table 2b). Three studies reported significant effects: (1) A significant decrease of BSI-18 scores was reported in a dyadic FTF PTP support setting (60–90 min.) in African BC patients compared to a brief telephone counselling (≤ 30 min.) as CG at 6 weeks and 6 months, but no significant difference was found at 12-month follow-up (Schover et al. 2011). (2) In patients with prostate cancer significantly lower GDS depression scores associated with the dyadic FTF PTP support at 2 months [M(± SD) = 2.53(± 3.662) and 0.92(± 1.320); *t* = 2.424, *p* = 0.02] were reported (Weber et al. 2007a). (3) A controversial effect was found in another study: patients with BC receiving an unmoderated OSG intervention showed a decrease in anxiety and depression measured with the HSCL-25 at 4 months, whereas patients in the CG improved in QoL (FACT-B scores) at 4 and 12 months post-intervention (time × condition, *p* = 0.05; *F* = 3.10; *df* = 76); the CG had reviewed information on a cancer-related website (Salzer et al. 2010). No benefits were detected for patients in the intervention group in terms of psychosocial outcomes (i.e., distress and QoL) at the 4- and 12-month follow-ups.

Seven studies assessed quality of life (Weber et al. 2007b; Sharif et al. 2010; Salzer et al. 2010; Schover et al. 2011; Osei et al. 2013; Giese-Davis et al. 2016; Toija et al. 2019) using various measures (2 × EORTC-QLQ-C30/EORTC-BR23], 2 × FACT-B, FACIT-SP, SF-12, UCLA) (Table 2c). Out of these, four studies reported significant outcomes for the intervention (TG): (1) BC-specific QoL/well-being [FACT-B] improved significantly more over time for patients in the TG vs CG, *t* = 2.41 ([95% CI 0.46–1.24], *p* = 0.02, *d* = 0.86) at 12 months. Interventions included either FTF, telephone-delivered support, or support via email (Giese-Davis et al. 2016). (2) Health-related QoL [EORTC-QlQ-C30] in patients with BC receiving FTF interviews improved significantly over time at 2 months follow-up (M(± SD), TG 80.0(± 17.9) vs. CG 61.66(± 21.88); time × group; *p* = 0.001). Additionally, significant improvement of cancer-related symptoms (i.e., fatigue, pain, insomnia, and loss of appetite, [*p* < 0.05], respectively) was reported for the TG. Further, a significant increase in all QlQ-BR23 dimensions was observed in TG (i.e., body image, sexual function, sexual satisfaction, and future perspectives [*p* < 0.001], respectively) (Sharif et al. 2010). (3) For patients with prostate cancer, significant improvements in prostate-specific QoL [UCLA] were reported at 2 months follow-up after dyadic FTF meetings: physical role functioning (physical role functioning (*p* = 0.01), bowel function (*p* = 0.03), mental health (*p* = 0.01), and social function (*p* = 0.04) (Weber et al. 2007b). (4) In another study in patients with prostate cancer (OSG intervention) the evaluation of the QoL measured by SF-12, EPIC-26 (Extended Prostate Cancer Index), Satisfaction with life scale, Relationship satisfaction scale, and Program satisfaction demonstrated significant improvements in three out of six areas: urinary irritation and obstruction health, sexual health, and hormonal health at 6 weeks (time × group [*F*(18) = 2.37, *p* = 0.036]), whereas at the 8-week follow-up, both groups returned to baseline level (Osei et al. 2013).

Five studies assessed other outcomes including psycho-sexual morbidity (Chambers et al. 2015, 2019), sexual functioning (Schover et al. 2011) trauma symptoms (Giese-Davis et al. 2016), and physical activity (Pinto et al. 2015; Galvão et al. 2018) (Table 2d). Significant differences associated with the intervention were reported in the two different interventions for physical activity (PA): (1) PA counselling provided by volunteers of BC survivors via 1t1 telephone contact showed a significant benefit in moderate-to-vigorous PA (MVPA) after 3 and 6 months [M(± SD), TG 103.0 min/week(± 15.4), *p* < 0.001; and CG 34.7 min/week (± 15.5, *p* = 0.03)]. Mean difference TG vs CG accelerometer MVPA data 48.5 min/week at 12 weeks (*SE* = 11.9) and 38.7 min/week (*SE* = 12.0) at 24 weeks (Pinto et al. 2015). (2) Compliance with exercise guidelines (i.e., resistance exercise duration) was evaluated in patients with prostate cancer, who received a telephone-delivered intervention led by two peer support volunteers for 6 months. A significant higher percentage of patients in the TG followed the exercise recommendations at the 3-month follow-up (time × group, *p* = 0.048, [95% CI 15–31.07]) and showed a significant longer duration of resistance exercise training at 3- and 6-month follow-ups (19.4 min/week [95% CI 6.52–32.28], *p* = 0.003, and 14.6 min/week [95% CI 1.69–27.58], *p* = 0.027) (Galvão et al. 2018).

Regarding the three PTP settings, dyadic telephone 1t1 was evaluated in *n* = 9 studies, dyadic FTF in *n* = 4, and web-based online support (OSG) in *n* = 4 studies. The effectiveness of the PTP settings cannot be compared with each other; nevertheless, significant effects were reported in *n* = 10 dyadic interventions (1t1 telephone-based, *n* = 5; dyadic FTF, *n* = 5) and in *n* = 2 OSG. Interventions using dyadic settings are compared to care as usual (in 11 of 12 studies), except for Schover et al. who investigated an FTF dyadic-intervention compared to a brief dyadic telephone counselling initiated by the participant (Schover et al. 2011). Regarding the control condition in the 4 OSG, information on cancer-related websites or written educational material was given, except for Klemm et al. who used a moderated OSG by social workers compared to a peer-led OSG (Klemm 2012).

We present an overview of all primary study outcomes, separated by PTP support setting and respective outcomes in Table 2a–d.

## Discussion

This review examined the effect of non-professionally led PTP support in cancer patients with respect to various cancer patient-related outcomes. Based on our inclusion criteria, we identified *n* = 18 RCTs (search from 2000 to 2023) focusing patients with breast cancer, prostate cancer, or mixed cancer types. Due to the heterogeneity of designs, measures and types of PTP interventions, we were not able to pool data for a meta-analysis. As types of PTP, most studies (*n* = 14) investigate dyadic 1t1 interventions by telephone or face to face, and *n* = 4 studies web-based online support.

Benefits associated with the PTP intervention were found in particular in patients with BRCA and prostate cancer, in FTF settings, and in the assessments of cancer-specific QoL outcomes. Most of the benefits sustained for a short period (4 weeks to 3 months). Guidance on how to prolong or maintain short-term effects is provided by Schover et al. who suggested that a workbook used to reinforce learning in both the interventional and control group condition accounted for most of the benefit (Schover et al. 2011). This requires further research to determine what measures can help sustain the short-term effects of PTP measures.

Our review shows that FTF peer support was detected as the most effective peer support format having positive effects in particular on self-efficacy, control, and knowledge related to cancer. Key aspects of FTF settings that might lead to benefits associated with the intervention are the individual setting, which creates a safe environment, which uses rituals, and provides commitment. This form of PTP support seems to allow the expression of genuine feelings. These results are in line with the recently published review of Ziegler et al. (Ziegler et al. 2022) or Hoey et al. (Hoey et al. 2008). As another important result of our review, patients benefit from PTP support especially in terms of cancer-specific QoL outcomes. These outcomes are covering aspects relevant to daily life of patients with cancer and may explain that significant effects of PTP support could be demonstrated as an improvement at the functional level or a reduction of physical complaints and symptoms (Weber et al. 2007b; Sharif et al. 2010). In addition, various studies detected significant improvement of psychological well-being and resilience capacity, reduction of depression, and/or anxiety or fear of recurrence as the most common issues patients are faced with. Providing PTP support can improve patients’ QoL, may enhance patients’ coping behaviour, reduce fear of cancer, and cultivate hope (Rini et al. 2014, 2007; Giese-Davis et al. 2016). In some studies, it is pointed out that peer-counselling should be provided by trained and supervised cancer survivors, but not in all studies, there is information if any type of specific training was a prerequisite for the peer support intervention. Although most of the studies are performed with cancer survivors whose initial treatment was completed, there is some evidence that PTP support by psychoeducation may be helpful already before and during ongoing treatment (Heydarzadeh et al. 2019).

Regarding various types of online support, our analysis revealed only a few significant effects of OSG with small or moderate effect sizes which are in line with the results of a Cochrane review focused on patients with BC, comparing OSG with a usual care group or comparing two or more types of OSG (i.e., forms of messaging [on a dedicated website or through email] or chat rooms). Pooled data from two studies (120 women with BC) showed a small to moderate reduction in depression, and pooled data from other two studies (140 women) showed no positive effects on QoL (McCaughan et al. 2017). A former systematic review on health outcomes of online-cancer support showed that none of the RCTs reported significant outcomes (Hong et al. 2012). Within the last year, across all entities, peers increasingly use online formats to support cancer survivors. Although OSG may be restricted compared to a personal FTF contact, online formats may allow a lively exchange between affected persons (Høybye et al. 2010). Further, on the common basis of personal concern, a feeling of togetherness develops that makes the exchange in the online forum particularly valuable for many users, which at the same time convey information, advice, and emotional support (Huber et al. 2018).

A review of peer supported RCT interventions on health and well-being across all health conditions and populations including 5% cancer patients observed beneficial effects only in a few trials (22 instances of 132 on mental health, and 28 of 113 on physical health outcomes); beneficial effects were reported across most outcomes, ‘most frequently with respect to behaviour change’ as promoting diet and exercise or smoking cessation (Campbell et al. 2004). The effects of support are not easy to investigate, because an RCT design to evaluate psychosocial outcomes is often not feasible without setbacks in study quality and constraints that patients have preferences and will therefore decline to participate in such research for fear of not receiving their choice of treatment (Solomon 2004). If they still participate in a randomized trial, it is to be suspected that empowered patients who are interested in enhancing their own well-being will use further sources of support.

From the methodological point of view, it was not surprising for us that we identified only a few RCT. In peer support, an RCT design is often not feasible due to rejection of randomization by the participants. Randomization contradicts with the basic principle of voluntary participation in PTP groups. Other barriers to RCTs include lack of blinding or uncontrolled confounders when usual care is used as the control group. In addition, a lot of influencing factors have to be considered. In the studies we reviewed, an analysis of specific parameters, which may influence the outcomes of PTP support as moderators or mediators, has not been done.

### Conceptual and methodological recommendations for future research

Common to all studies included in this review, PTP support provided by non-professional fellow patients (i.e., treatment group, TG) was compared to a control group (CG), except for one study that compared support by peers versus professional nurse support versus control (Chambers et al. 2015). As a methodological shortcoming, the characteristics of the CG were not described in detail in any of the studies included. It is unknown what resources the CG may have used beside the peer support. In most studies, this is not assessed, although in many countries, professional-led psychological and/or psychosocial support is often part of care as usual.

The analysis of relevant subgroups as moderators to the outcomes is understudied in most trials. Through not-harmonized precise support group definitions, it is further difficult to identify outcomes and influencing factors (Campbell et al. 2004; Hoey et al. 2008). Giese-Davis et al. who evaluated the effects of a dyadic telephone peer-counselling for women newly diagnosed with BC have analysed that the subgroup of highly traumatized patients improved more than non-traumatized and more than controls in terms of self-efficacy, depressive symptoms, and FACT-B BC-specific well-being (Giese-Davis et al. 2016). Women with genetically determined BC, who took part in a 1t1 telephone peer intervention, showed a significant reduction of distress compared with the controls (care as usual) (White et al. 2014).

Overall, the studies are extremely heterogeneous with respect to measurement and design. Various outcomes are used and assessed with a variety of measures which makes it difficult to compare. In addition, duration and frequency of the interventions as well as the time points of measurement vary across the studies. Therefore, the study quality and the level of evidence of PTP support in patients with cancer are low.

### Limitations

In our review analysing the effects of PTP support on various outcomes, we were faced with various flaws in terms of methodological rigor of the studies, e.g., lack of control for confounders, lack of blinding, and the variety of instruments used for measuring the outcomes. Therefore, the analysis of pooled data was not possible. The overall RoB rating demonstrates a mixed quality of the studies included. This means that we have to interpret these results with caution on the background of the quality assessments. Additionally, it has to be considered that we may have excluded studies in which PTP support was not explicitly described or the impact of professional support on the programm was not clear. Further, we searched only studies published in English and German, which may have excluded other studies published in another language.

### Clinical implications

Peer-to-peer support is an important part in non-professional patient care that contributes to improvements in cancer-specific QoL of patients. Therefore, PTP research should also focus the quality parameters of successful and efficient PTP support, e.g., standardized training for the peers guiding or moderating the PTP interventions.

## Conclusion

Peer-to-peer support is an important part in non-professional patient care and will increase in its relevance in the future. Further, participation of patients in all fields of health care and research is required by professionals, politicians, and stake holders (Solomon 2004; Gidugu et al. 2015). This requires a systematic and sustainable cooperation between professional health care facilities and patients’ support groups by involving patients’ representatives in various panels like patients’ advisory boards in clinics or steering committees of clinical trials.

For future research, we recommend to improve methodology and scientific rigor by (1) conducting RCT with clear defined control groups including long-term follow-ups and (2) exploring the effects of peer support in cancer patients using multilevel approaches and cancer-specific instruments. PTP research will also benefit from both quantitative and qualitative research methods to detect the benefits of various types of PTP support (i.e., individually, in groups both digital and personal). More studies with other diagnostic groups than Breast and Prostate cancer are needed.

## Data Availability

The authors confirm that all data used in this review are included in this published article. Furthermore, primary and secondary sources and data supporting the findings of this study were all publicly available at the time of submission.

## Appendix

Risk of Bias Figures. (See Figs. 2 and 3)
